# Cobalt and nickel ion synergy promotes gene amplification and stable metal resistance in *Shewanella oneidensis*

**DOI:** 10.1128/aem.01844-25

**Published:** 2026-07-10

**Authors:** Alessandra G. Gavin, Stephanie L. Mitchell, Erin E. Carlson

**Affiliations:** 1 Department of Chemistry, University of Minnesota 172305, Minneapolis, Minnesota, USA; 2 Department of Medicinal Chemistry, University of Minnesota 731060, Minneapolis, Minnesota, USA; 3 Department of Biochemistry, Molecular Biology, and Biophysics, University of Minnesota 200766, Minneapolis, Minnesota, USA; 4 Department of Pharmacology, University of Minnesota 214458, Minneapolis, Minnesota, USA; Washington University in St Louis, St. Louis, Missouri, USA

**Keywords:** resistance evolution, nickel, cobalt, *Shewanella oneidensis*, gene amplification

## Abstract

Microbial species are integral in environmental homeostasis and have thus developed diverse strategies to adapt, survive, and proliferate under exogenous stress. The growing demand for nanoscale battery materials has led to increasing environmental concentrations of nanomaterials and their constituent metal ions. Previous work has shown that the Gram-negative bacterium *Shewanella oneidensis* is able to rapidly evolve resistance to one such nanomaterial, lithiated nickel manganese cobalt oxide (NMC). Yet, the specific stimuli that trigger resistance evolution and the ensuing genomic changes were previously unknown. Here, we demonstrate that the combination of cobalt and nickel ions released from NMC trigger a gene amplification event that enables stable nanomaterial and metal resistance, likely caused by amplification of a cation efflux pump. Amplification copy number is highly dynamic over the period of study, yet amplification events persist over prolonged recovery in the absence of metal stress. Growth rate comparisons reveal no physiological cost associated with a high copy number. The stability of this genomic mutation combined with the lack of observable fitness cost distinguishes this genomic perturbation from previously reported gene amplification events. Finally, the observed amplification event was unique to the combination of cobalt and nickel, which implies that the intracellular targets of these metal ions have a specific interaction that yield resistance. Ultimately, we report a dynamic and specific gene amplification event that allows *S. oneidensis* to survive metal stress. This work not only illuminates the broader ecological consequences associated with introducing nanomaterials and metals into the environment but also provides insight into the larger scope of bacterial resistance mechanisms.

**IMPORTANCE:**

Studying mechanisms of microbial resistance evolution is critical for understanding the role of bacteria in the environment and developing new strategies to combat antimicrobial resistance to modern therapeutics. Technological innovation has often led to the introduction of novel environmental stressors that impart pressure on microbes to evolve resistance. This may ultimately lead to novel resistance genes or gene cassettes in the environmental gene pool, which could further promote antimicrobial resistance in pathogenic organisms. In this study, we highlight the importance of considering gene amplification as a mechanism of bacterial resistance to environmental toxins. We demonstrate that amplification events can exist within a population in the absence of selection pressure and without a clear fitness cost. Identifying specific stimuli that trigger these events will help us understand factors that accelerate bacterial resistance evolution and have the potential to disrupt ecosystem balance.

## INTRODUCTION

Bacterial species are integral components of the environmental microbiome and are known to rapidly adapt and evolve under selective pressure (1). This plasticity allows bacteria to temporarily survive in diverse and hostile environments and creates the opportunity for cells to acquire beneficial genomic mutations that promote sustained survival against specific stressors (2–5). Yet, the mutations that facilitate bacteria survival often come at the cost of fitness and thus may impact the composition of microbial communities (6, 7). The cost is often worth paying as these adaptive strategies have enabled bacteria to develop resistance to a diverse array of antibiotics and other toxins, including engineered nanomaterials (ENMs) and metal ions (8). The use of ENMs in consumer goods has increased dramatically over the past two decades, and has resulted in a detectable increase in the prevalence of ENMs and associated raw materials in waste streams and the environment (9). Although many of these materials are ecologically benign, some ENMs and associated metal ions have demonstrated toxicity to a variety of organisms (10, 11).

Nanoscale lithiated nickel manganese cobalt oxide (NMC) is a commonly used cathode material in electric vehicle batteries and research has established NMC toxicity towards bacterial species. NMC toxicity stems from a combination of reactive oxygen species (ROS) and cobalt and nickel metal ions released from the metal-oxide framework in an aqueous environment (12). Furthermore, challenges associated with developing large-scale recycling infrastructure makes environmental release of NMC inevitable (13–15). Evaluating bacterial response to ENM stress is not only critical for understanding the ecological impact of environmental toxins but also furthers our understanding of fundamental mechanisms of bacterial adaptation and evolution.

*Shewanella oneidensis* is a ubiquitous, facultative anaerobic γ-proteobacterium known for its role in environmental metal cycling. *S. oneidensis* is found in diverse aquatic environments, including sediment and soil, which makes it likely that *S. oneidensis* will be present in nanomaterial-contaminated environments (16–18). We have previously demonstrated that chronic exposure to NMC or associated metal ions causes rapid and stable resistance in *S. oneidensis* (19). Understanding the physiological changes associated with metal and NMC resistance is critical due to the role of *S. oneidensis* in environmental metal cycling and maintaining environmental homeostasis (20, 21). By understanding how exogenous stressors fundamentally change *S. oneidensis*, we can more clearly assess the environmental consequences of metal and nanomaterial pollution.

When bacteria first encounter exogenous stress, cells can activate a general stress response to increase transcriptional regulation of protective genes (22, 23). This initial regulatory response can have a dramatic effect on bacterial physiology and is often accompanied by reduced growth rate to enable short-term survival, but does not necessarily promote sustained growth under stress or resistance evolution (24–26). Notably, when cells are removed from direct stress, they lose these specific stress adaptations and return to being phenotypically and genotypically identical to wild-type (WT) cells (27). Under chronic stress, cells may develop and select for mutations in specific gene(s) or pathways that enable stable and heritable resistance to stress with limited physiological costs (28). Bacteria adapt and resist metal stress through a variety of cellular mechanisms including metal sequestration, changes in cell permeability, alteration of metal target sites, enzymatic detoxification, and changes in efflux machinery (29–33). In the context of metal resistance, however, beneficial gene mutations can take thousands of generations to develop and dominate the population. The slower nature of resistance gene evolution often promotes intermediate adaptation mechanisms that enable survival in the interim. Chromosomal mutations, such as gene duplication amplification (GDA) events or the mobilization of mobile genetic elements (MGEs), can serve as this adaptive intermediate tolerance mechanism (1, 34).

GDA events can occur within 100 generations of toxin exposure and may involve 2- to 100-fold amplification of specific genes within the bacterial chromosome (25). Furthermore, the kinetics of GDA events are stochastic, and copy number can vary significantly within a population (35). The size of the amplified region is also highly variable, with reported amplified regions ranging from 18 to 300,000 bp (8, 25). Various stressors, including carbon source, antibiotics, and heavy metals, are known to select for high copy number variants, yet the mechanisms by which GDA events are initiated are poorly understood (26, 36–40). Importantly, the presence of MGEs in a bacterial genome can facilitate gene amplification in response to exogenous stress (41). MGEs promote tolerance to toxins through either increased copy number of beneficial genes or insertion into new regions of the genome and subsequent disruption of native genomic function (42). It is typically observed that cells with higher copy numbers (i.e., more amplification events) are more resistant to a given stimulus; however, organism fitness generally decreases as the bacteria maintains these additional copies. It is thought that this genomic redundancy presents the opportunity for beneficial mutations to develop and propagate while protecting the organism from catastrophe if a mutation develops in an essential gene. Thus, high gene copy number protects the genome from harmful mutations while providing resistance to a given stress to allow for further evolution of resistance genes that become dominant in a population without an associated fitness cost (28, 43).

Gene amplification events occur rapidly to enable survival under in specific stress conditions, but the transient nature of gene amplification and associated fitness costs have led to the exclusion of gene amplification from discussions of stable mechanisms of bacterial resistance. Our work demonstrates that a stable gene amplification event in *S. oneidensis* evolves during exposure to cobalt and nickel and enables stable NMC and metal resistance. More specifically, this gene amplification is a consequence of the unique combination of cobalt and nickel metal ions. Contrary to other studies evaluating gene amplification events, there is no clear fitness cost associated with metal-induced amplification in *S. oneidensis,* and, although copy number decreases over time, amplifications (>1) persist for over a thousand generations in the absence of metal stress. No common mutations were observed with prolonged metal stress, which further indicates that amplification may serve as a primary, stable, mechanism of multi-metal resistance in *S. oneidensis*. Thus, the work presented herein reaffirms the need to evaluate gene amplification as a stable mechanism of bacterial resistance. The importance of understanding the dynamics of gene amplification events in metal resistance is underscored by the growing body of literature linking metal resistance to the environmental dissemination of antibiotic resistance genes (44–46).

## RESULTS

### Gene amplification is a consistent DNA perturbation associated with metal resistance

Previous work from our group has demonstrated that metal ions drive stable and hereditable resistance evolution to NMC nanomaterial in *S. oneidensis* (19). The implication of stable and hereditable resistance to metals and NMC is that chronic metal exposure triggers a genome-level alteration. To interrogate genomic perturbations associated with resistance, cells were subject to whole-genome sequencing (WGS). A total of 23 NMC-resistant colonies and 10 metal-resistant colonies from four separate resistance evolution experiments were submitted for WGS. Mutations in specific cellular machinery associated with metal sequestration, efflux, or enzymatic detoxification are common drivers of metal resistance in bacterial cells. However, WGS did not reveal any mutations in metal-related systems after either metal- or nanomaterial-resistance evolution (Table S1). Unsurprisingly, NMC-exposed cells had a higher mutation frequency than metal-exposed cells, which is likely due to additional ROS generated by the nanomaterials relative to metal ions (12). Seven of the 23 sequenced NMC-resistant colonies had genomic mutations. These mutations corresponded to the following genes: ribosomal protein *rpsK*, RNA polymerase *rpoA*, outer membrane porin, flagellar structural component *flgB*, and three distinct non-coding regions. Furthermore, only one of the 10 sequenced metal-resistant strains had a mutation. The observed mutation was a frameshift in a hypothetical protein located on *Shewanella’s* mega plasmid and thus was similarly not in a gene previously associated with metal resistance. The disparate nature of the mutations observed in NMC- and metal-resistant *S. oneidensis* indicates that consistent resistance evolution is likely driven by an alternative genetic perturbation.

**Fig 1:**
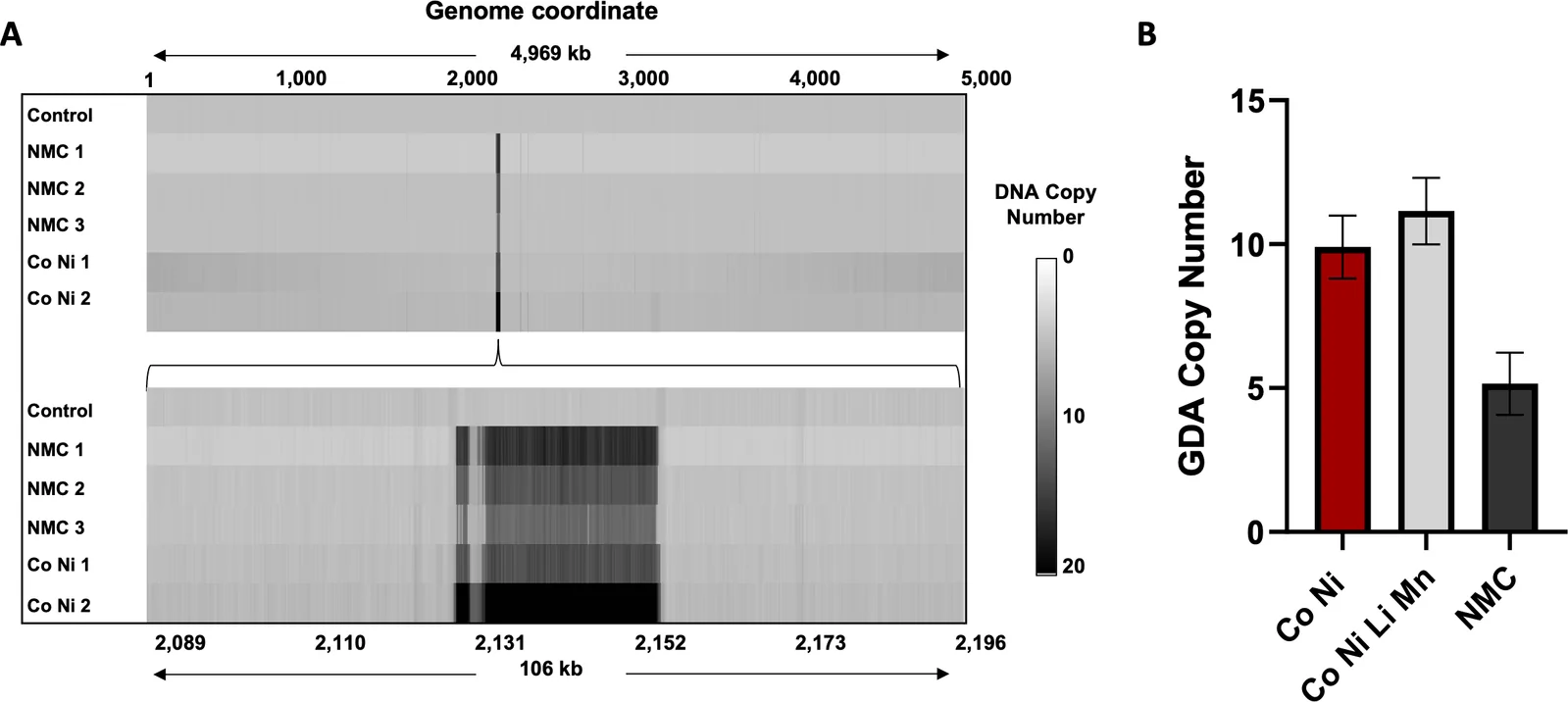
Genomic sequencing and gene copy number analysis for NMC and metal-resistant strains. (**A**) Whole-genome sequencing read coverage depth of control and NMC-exposed strains. (**B**) qPCR analysis of copy number in cells with evolved resistance to cobalt and nickel, all ions (cobalt, nickel, manganese, and lithium), and NMC.

Further analysis of WGS revealed an increase in read coverage over a 28,333 bp region of the genome (i.e., coordinate 2,128,886 through 2,157,241) in all metal and NMC-resistant strains. Gene coverage maps indicate that this region of interest (ROI) is amplified between 10 to 25 times after only 48 generations of stress exposure in minimal media. Notably, no evidence of amplification events was found in WT or passaged control strains (Fig. 1A). Previous work has demonstrated that metal ion exposure is sufficient to elicit resistance phenotypes in *S. oneidensis*, and here, we demonstrate that resistance to metals and nanomaterials causes the same genetic amplification (Fig. 1A and B). This suggests that metal exposure drives resistance evolution. Putative annotations of the genes within this ROI are listed in Table S2. Interestingly, the ROI is flanked by transposases, indicating a potential mobile genetic element (41).

Due to the presence of both flanking transposases and integrases, it is necessary to consider the possibility that the amplified region is translocated to new region(s) of the genome. Long-read sequencing was conducted to evaluate where duplication events were inserted in the genome (47). To determine if the ROI was mobilized to new locations, we specifically analyzed reads that map to multiple locations in the genome (split reads) and cover at least half of the region of interest (coordinates 2,128,886 to 2,157,241). We then sorted these reads for tandem repeats, both ends of the split read map to the ROI, and distal translocations, one end of the split maps to the ROI and the other maps >10 kb away from the ROI. Examining the read count in sequenced strains for each condition, we see that tandem repeats are noticeably more common than distal translocation (Table S3). The resulting split reads were also visualized in a dot plot, which further demonstrated that for the large majority of junction reads, both ends map back to the ROI (Fig. S1). It should be noted that we did detect instances of distal translocations; however, these insertion events occurred with much lower frequency than tandem repeats and were detected with the same frequency in metal-resistant strains and passaged control. Furthermore, inconsistent insertion locations among the reads that map to distal locations suggest that resistance is driven by a high copy number of cargo genes rather than translocation landing site. These data do not disprove the possible translocation of cargo genes but suggest that the primary structural variation involves back-to-back duplication of the ROI. The resulting long-read sequencing suggests that duplication copy number rather than insertion location or mutation in a single gene drive NMC and metal resistance in *S. oneidensis*.

### Cobalt and nickel synergy drive DNA amplification and resistance evolution

**Fig 2:**
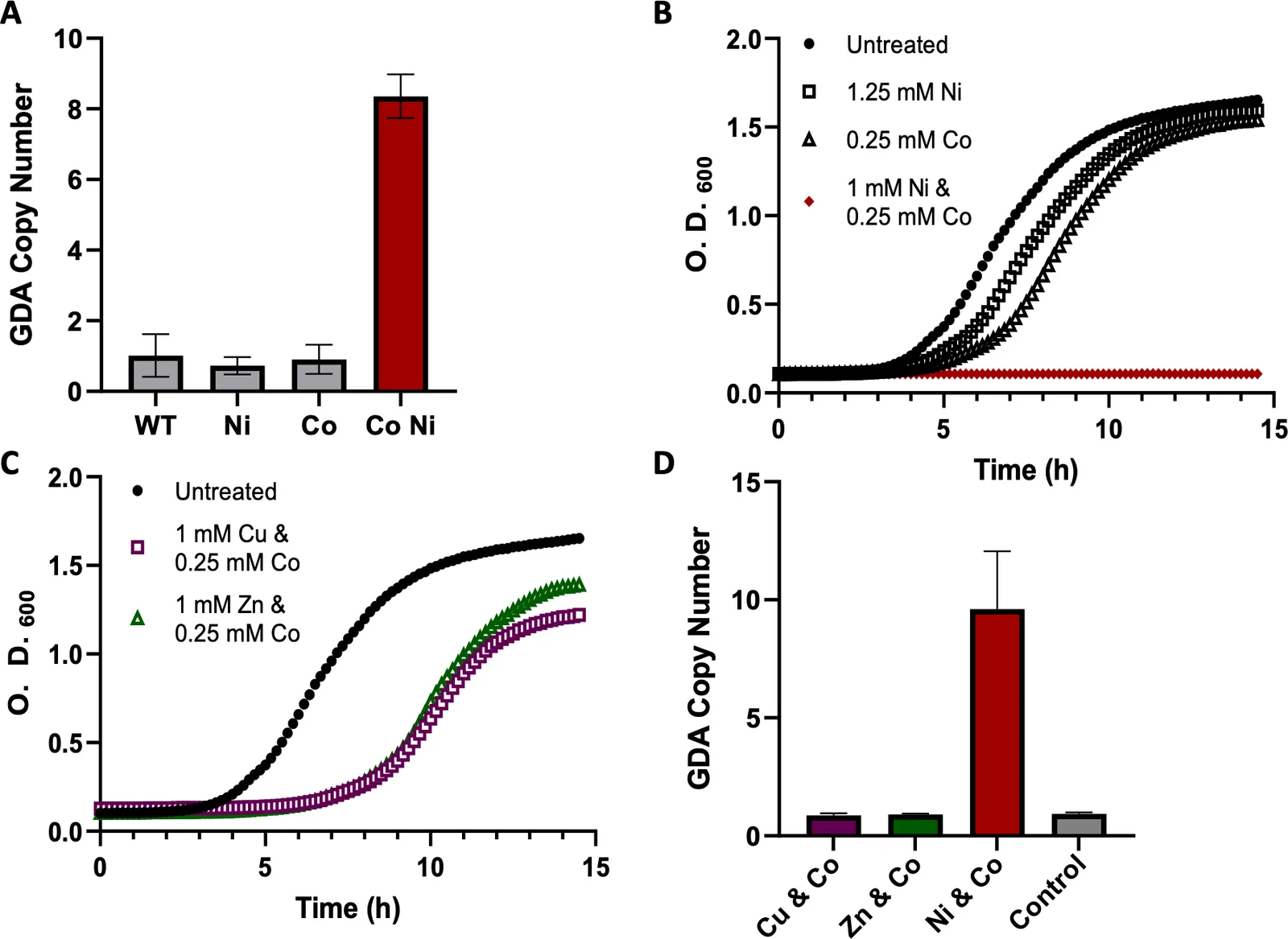
The specific combination of cobalt and nickel interacts synergistically to facilitate gene amplification-mediated resistance in *S. oneidensis*. (**A**) qPCR analysis of DNA copy number upon individual and combination ion exposure after 48 generations in minimal media. (**B**) Bacterial growth curves with mono and co-treatment of cobalt and nickel metal ions. (**C**) Co-treatment cobalt and either zinc or copper. (**D**) qPCR analysis of DNA copy number after 120 generations in LB with various combinations of metal ions.

Because amplification events are observed in both metal- and nanomaterial-resistant cells, we sought to determine the chemical identity of the stressor(s) that drive gene amplification. Previous work has demonstrated that the combination of cobalt and nickel metal ions most closely mimic NMC toxicity, and lithium and manganese impart no additional toxicity to *S. oneidensis* (48). As such, we evaluated if concentrations of cobalt and nickel ions equivalent to that released from NMC in a defined minimal medium (25 µM nickel and 10 µM cobalt) were sufficient to elicit copy number amplification. Upon exposure to cobalt and nickel for 48 generations, copy number was equivalent to that of cells exposed to the combination of all ions released from the NMC framework (lithium, nickel, manganese, and cobalt) or nanomaterial itself in minimal media (Fig. 1B). This prompted further investigation into the individual contributions of cobalt or nickel metal ions on copy number evolution. In subsequent experiments, copy number was evaluated through a qPCR-based method involving two primer sets, one for a gene within the amplified region (*SO_2041*) and one for a single-copy gene outside the amplified region (*SO_4604*) (Table S4) (49). It should be noted that this qPCR-based method uses a single point as a proxy for the whole region of interest and provides no information about how this region may be moving in the genome. Interestingly, no amplification of the ROI was observed over 48 generations with individual metal exposure (10 µM cobalt or 25 µM nickel), whereas approximately eight amplifications were observed with 35 µM metal combined treatment (10 µM cobalt and 25 µM nickel) over the same 48-generation timescale (Fig. 2A). This implies that the co-exposure to cobalt and nickel metal ions has a synergistic effect on amplification. To ensure that amplification is dependent on metal species rather than metal concentration, we exposed *S. oneidensis* to higher concentrations of individual metal ions (35 µM cobalt or nickel). Similarly, no duplication events were observed (Fig. S2). These results indicate that the combination of cobalt and nickel metal ions is necessary and sufficient to elicit rapid and specific gene amplification in *S. oneidensis*.

To further profile the specific interaction of cobalt and nickel in *S. oneidensis*, we evaluated individual and combined toxicity of cobalt and nickel to WT cells. We sought to determine if the combination of cobalt and nickel was additive or synergistic to better understand how specific metal stress drives gene amplification. Individual and combined metal MICs in nutrient-rich media were established using a checkerboard assay and synergy was then evaluated by calculating the fractional inhibitory concentration (FIC) value. FIC is used to quantify the change in toxicity between a single compound and multiple compounds to determine if the interaction of the two toxicants is antagonistic, additive, or synergistic. FIC is calculated by comparing the MIC of individual toxins to that of toxins in combination (50). The concentration of metal ions used for these assays ranged from 0.25 to 4 mM nickel and 0.125 to 2 mM cobalt. The MIC values of nickel and cobalt ions in nutrient-rich media were 4 and 2 µM, respectively, whereas the MIC for these metals in combination was 1 µM nickel and 0.25 µM cobalt (Fig. 2B). The calculated FIC value was 0.375, indicating that nickel and cobalt toxicity is synergistic in *S. oneidensis*:


$$Fractional\;Inhibitory\;Concentration\;(FIC)=\frac{MIC_{Co(Co+Ni)}}{MIC_{Co}}+\frac{MIC_{Ni(Co+Ni)}}{MIC_{Ni}}$$

We postulate that the synergistic interaction of nickel and cobalt facilitates DNA amplification and resistance evolution. To our knowledge, this is the first report of cobalt and nickel synergy in bacterial cells.

### Metal synergy and gene amplification is unique to cobalt and nickel

The strong synergistic interaction between cobalt and nickel in *S. oneidensis* prompted us to investigate potential synergy with other first row transition metals. The toxicity of copper and zinc metal ions in nutrient-rich media was found to be equivalent to that of nickel (minimum inhibitory concentration equal to 4 mM). Because of this similarity in individual metal ion toxicity, we substituted either copper or zinc for nickel and repeated the previously described checkerboard assays to evaluate potential synergistic toxicity between alternate metals. Interestingly, no novel combination of zinc or copper with cobalt demonstrated synergistic toxicity in *S. oneidensis* (Fig. 2C). Despite the clear lack of metal synergy, we exposed cells to the combination of alternate metal ions (i.e., 0.25 mM cobalt and 1 mM of either copper or zinc) for ~120 generations in Lysogeny Broth (LB) looked for the evolution of gene amplification events within the population. Interestingly, we found that exposure to either copper and cobalt or zinc and cobalt yielded no gene duplications, whereas the combination of nickel and cobalt gave rise to between 8 and 13 amplification events (Fig. 2D). These results indicate that the intracellular interaction between cobalt and nickel is highly specific, and it is unlikely that the observed gene amplification is a general stress response but rather a coordinated response to specific stimuli.

Interestingly, cells exposed to the combination of copper and cobalt or zinc and cobalt for 120 generations still had a prolonged lag phase indicating that cells did not adapt to metal stress over this time (Fig. S3). This implies that *S. oneidensis* cannot rapidly adapt to these alternate metal combinations through the same mechanism that allows for resistance to cobalt and nickel. These results indicate that DNA amplification is a unique response to the combination of cobalt and nickel, and the cell does not have an equivalent duplicative mechanism to deal with toxicity from other combinations of metal ions.

### Cells maintain elevated copy numbers and metal resistance in the absence of metal stress

Considering the ecological implications of metal resistance, we sought to determine if elevated copy numbers persist in *S. oneidensis* after an extended recovery period without metal stress. We have previously demonstrated that cells retain metal resistance after ~60 generations in media devoid of metals, which suggests that genetic perturbations persist for multiple generations. However, other work that has looked at gene amplification events demonstrates that amplifications are lost rapidly once stressors are removed. Cells are thought to lose most additional copies within 200 generations without stimuli.

To explore long-term stability of the accumulation of amplification events, we passaged metal-resistant cells (copy number ~10) in LB media without metals for approximately 1,128 generations and evaluated copy number throughout. Interestingly, there was a rapid increase in copy number from ~6 to 15–20 copies over the first 50 generations. This rapid increase was followed by a steady decrease over the next 350 generations to a copy number of ~5. After 400 generations, copy number continued to slowly decrease but stabilized at 2–3 copies, and most notably does not return to one copy over the course of our experiment. These data indicate that although copy number decreases, it does not return to one after 1,128 generations in the absence of metals.

**Fig 3:**
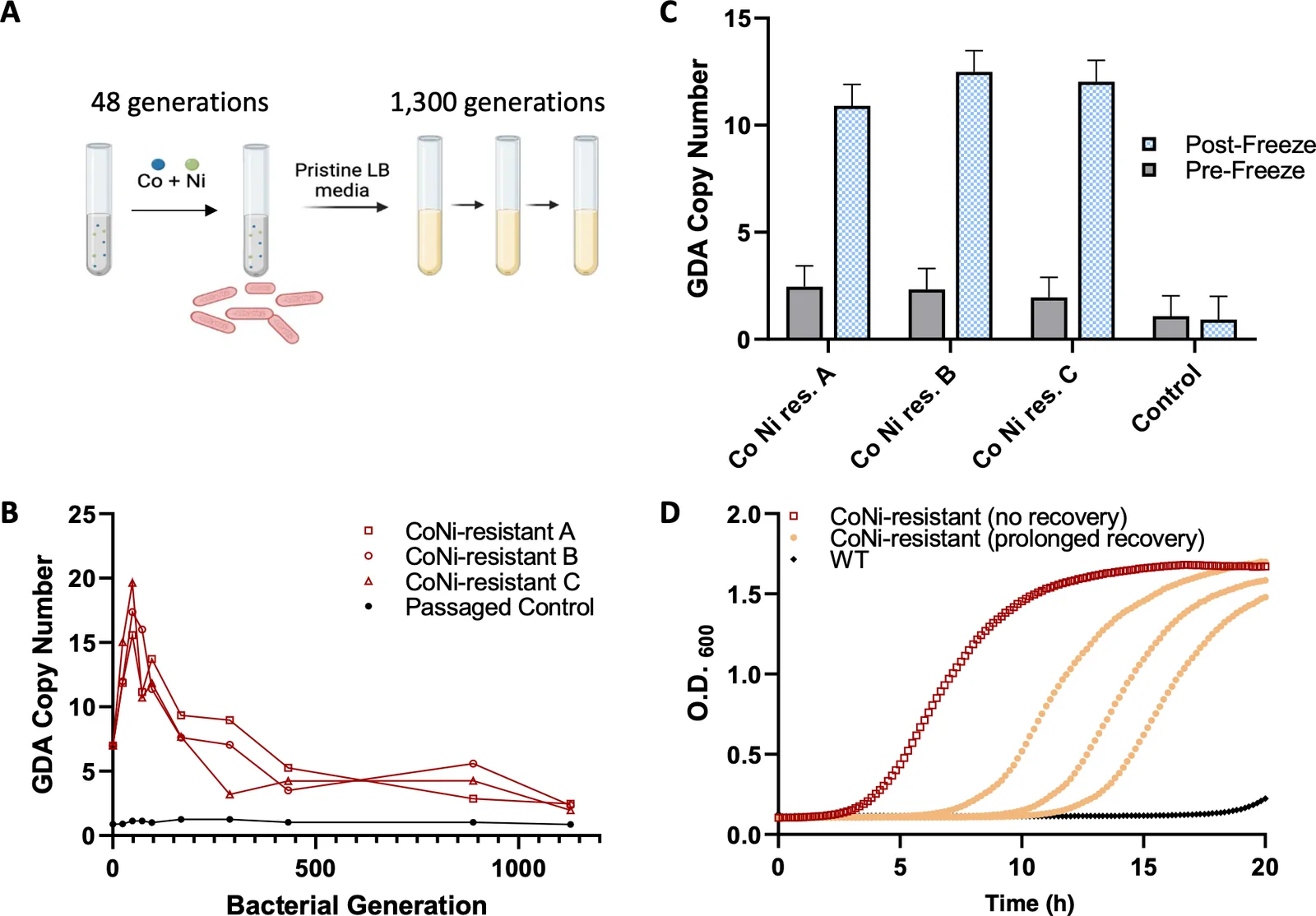
Gene amplification dynamics of metal-resistant cells over 1,128 generations without metal stress. (**A**) Experimental scheme of resistance evolution and cellular recovery. One passage corresponds to 24 generations. (**B**) qPCR analysis of gene copy number in three biological replicates over multiple time points throughout the ~1,100-generation recovery period. (**C**) qPCR analysis of gene copy number after one freeze-thaw cycle following a 1,128 generations. (**D**) Bacterial growth with metals in cells that have and have not experienced a prolonged recovery period in nutrient-rich media.

Finally, we evaluated metal tolerance in resistant cells after 1,128 generations with no metal stress. Cell growth was monitored over 20 h in LB with 1 mM nickel and 0.25 mM cobalt. This concentration of metals is lethal to WT cells but not to resistant cells. We found that metal-resistant cells that underwent prolonged recovery were able to grow with notably shorter lag phases than WT cells exposed to metals, and growth rate was nearly identical to WT cells with no metal exposure (Fig. 3D). Together, these data indicate that cells retain metal resistance after prolonged recovery. To determine if compensatory mutations evolved over 1,128 generations devoid of metal stress, three strains were subjected to whole-genome sequencing. Unsurprisingly, additional mutations were detected after 1,128 generations; however, no mutations were found in common cellular components or pathways (Table S5). Furthermore, the detected mutations were in various intergenic regions, suggesting that these mutations are a byproduct of prolonged culturing and likely not advantageous. These sequencing data further support gene amplification as a primary mechanism of metal resistance in *S. oneidensis*.

It was our original intent to use the cells that underwent a prolonged recovery period to study the impact of low copy number on metal resistance. Thus, cells were made into glycerol stocks for future use. Interestingly, when these stocks were streaked on agar plates and grown in pristine media, we found that copy number immediately jumped back up to ~10 in all metal-resistant replicates (Fig. 3C). It is important to note that passaged control cells did not have additional copies after cryopreservation, which implies that cryostress does not induce duplication unless there is a prior history of stress. This surprising increase in copy number after cryopreservation highlights the dynamic nature of gene amplification in metal-resistant *S. oneidensis* and the preference cells have for retaining amplification events once they arise. Finally, although freeze-thaw does not induce gene amplification in passaged control cells, changes in copy number observed in metal-resistant cells suggest that other stressors may induce amplification events, which is an important consideration for future work.

### Metal-induced gene amplification has no inherent fitness cost in LB media

**Fig 4:**
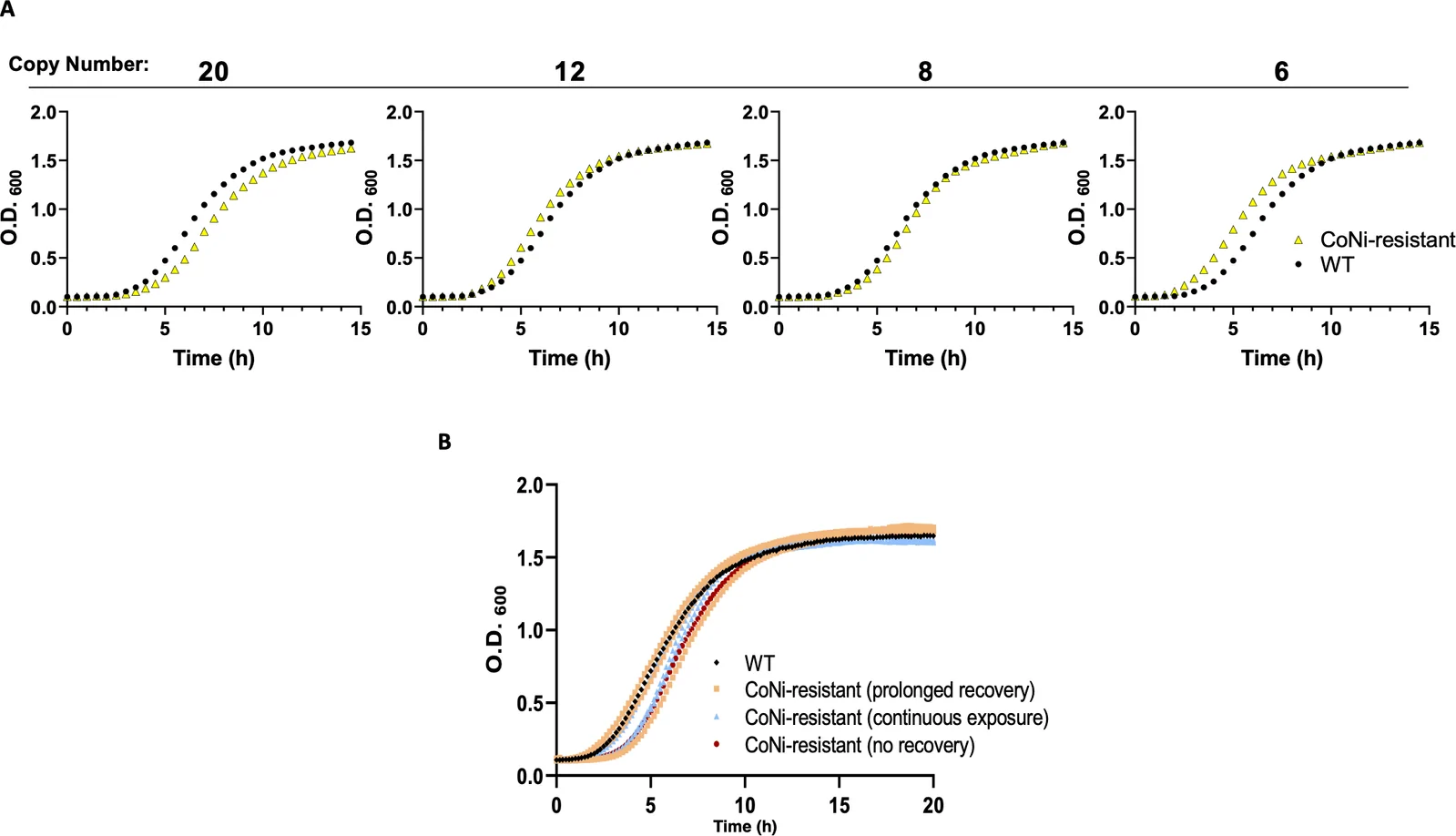
Bacterial growth curves in nutrient-rich media without metal stress. (**A**) NMC-resistant cells with between 6 and 20 amplification events. (**B**) Metal-resistant cells after either 1,128 generations of recovery (CNR_PR), 1,344 generations of continuous metal exposure (PMT), no recover period, or untreated control (WT).

It has been reported that each additional kb of DNA results in a 0.15% reduction in cell fitness (51). Thus, a 10× amplification event of 28.3 kb in *S. oneidensis* would equate to a 4.25% reduction in fitness. Copy number in metal and NMC-resistant cells is heterogeneous, which suggests that cells have imperfect control over amplification and begs the question of whether there is an observable difference in organism fitness based on copy number. Specific growth rate (µ) and lag phase duration in LB media were evaluated in NMC and metal-resistant strains without metal stress. These cells had between 6 and 20 amplification events yet had indistinguishable growth dynamics in LB media (Fig. 4A; Table 1). It should be noted that the use of LB media for growth dynamic experiments could mask small differences in cell growth that may be observed in a minimal media (52). Although we cannot detect minute differences in growth rate, we can determine that the copy number does not have a detrimental effect on cell growth.

**TABLE 1:** Gene amplification copy number and growth metrics (specific growth rate [µ] and lag phase) in various strains of NMC-resistant *S. oneidensis*

Strain	Copy number	Specific growth rate (µ)	Lag phase (h)
Wild-type	1	0.6414	2.5
NMC 1	12	0.529	3.5
NMC 2	6	0.639	2
NMC 3	8	0.661	2.5
NMC 4	13	0.489	2.5
NMC 5	10	0.640	2.5
NMC 6	20	0.602	2.5
NMC 7	9	0.704	2
NMC 8	12	0.614	1.5

Further growth rate analysis was conducted in metal-resistant cells after a prolonged recovery period and extended metal exposure (discussed below). Growth dynamics (specific growth rate and lag phase duration) was nearly identical in LB media across all strains of *S. oneidensis* regardless of metal exposure or recovery duration (Fig. 4B; Table 2). This further supports our initial observations that copy number does not impact cell fitness under laboratory conditions.

**TABLE 2:** Gene amplification copy number and growth metrics (specific growth rate [µ] and lag phase) in metal-resistant *S. oneidensis* after extended recovery, extended metal exposure

Strain	Copy number	Specific growth rate (µ)	Lag phase (h)
Wild-type	1	0.642	1.5
Extended exposure 1	1.42	0.685	2.5
Extended exposure 2	0.96	0.530	1.5
Extended exposure 3	4.7	0.6221	1.5
Extended recovery 1	7.01	0.667	2.5
Extended recovery 2	9.77	0.635	2.5
Extended recovery 3	5.79	0.602	1.5

### No clear compensatory mutations develop over 1,000 generations of metal exposure

Because gene duplication amplification events are often described as transient mechanisms of bacterial adaptation, we were interested in probing gene amplification dynamics and evaluating potential compensatory mutations after extended metal exposure (i.e., >1,000 generations with metal stress). *S. oneidensis* routinely acquired between 7 and 20 amplification events after 48 generations of metal exposure (25 µM nickel and 10 µM cobalt) in a defined minimal media (19). We previously demonstrated that cells have fully evolved metal resistance after 48 generations, meaning that specific growth rates are identical with and without metal stress. Considering the variability in amplification frequency, we were interested in the dynamics of amplification over thousands of generations of metal exposure. Because growth rate of *S. oneidensis* in minimal media is slow (~6 h doubling time), metal exposure was adapted for nutrient-rich LB, (~30-min doubling time).

**Fig 5:**
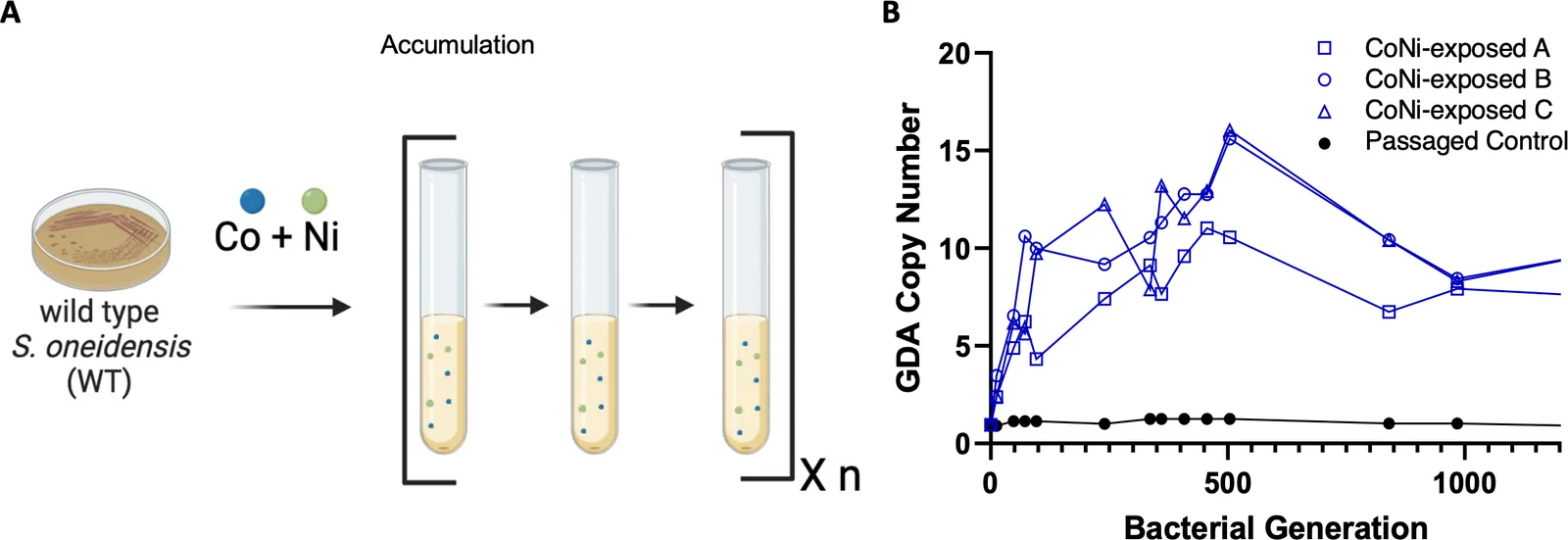
Copy number accumulation over extended duration of metal exposure. (**A**) Experimental scheme. (**B**) qPCR analysis of copy number at various timepoints throughout 1,344 generations of exposure to the combination of 1 mM nickel and 0.25 mM cobalt in three strains of *S. oneidensis* and a parallel passaged control.

We first optimized metal concentration to find a similar toxicity in LB as previously observed in minimal media (19). It was found that 0.25 mM cobalt and 1 mM nickel were lethal to *S. oneidensis* in LB, but chronic exposure to this concentration of metal ions in LB led to the evolution of metal resistance in a manner consistent with what is observed in a minimal media. Thus, LB broth was used for all subsequent studies presented in this work. Cells were then passaged for approximately 1,344 generations in LB with 0.25 mM cobalt and 1 mM nickel. Copy number was analyzed at various time points and found to increase rapidly over the first 72 generations from one to ~10. After 72 generations, amplification events continued to accumulate before reaching a maximum copy number of ~15 by generation 500. Copy number ultimately decreased and equilibrated around 10 after an additional 700 generations of metal exposure (Fig. 5B). These results demonstrate that copy number accumulation is dynamic, but cells retain 10 or more copies with prolonged metal stress.

Previous analysis of gene duplication amplification events demonstrates that cells often acquire compensatory point mutations after just 100 generations of continual stress (28). Ultimately, these compensatory mutations became the dominant mechanism of resistance, and unnecessary copies of gene amplifications were lost in the population. To investigate whether 1,344 generations of metal treatment led to the evolution of compensatory point mutations, we sequenced three strains that underwent prolonged metal treatment. The sequenced strains had no common mutations, and interestingly, one of the three sequenced strains had no genomic perturbations other than amplification of the 28.3 kb region (Table S6). The mutations found in the other sequenced strains include a single nucleotide polymorphism (SNP) in a cyclic AMP response regulator (*SO_0624*), an insertion in the intergenic region between *SO_1117* and *SO_1120*, and a SNP in adenylate cyclase*, cyaC*, and iron oxygenase, *SO_1944*. The lack of common compensatory mutations is further supported by evidence that cells retained ~10 amplifications after 1,344 generations of metal treatment (Fig. 5B), and that all strains have similar fitness to untreated *S. oneidensis* in the absence of metal stress (Fig. 4B). Taken together, these observations suggest that gene amplification is the predominant mechanism of cobalt and nickel resistance in *S. oneidensis*.

### Putative cation efflux pump is necessary for cobalt and nickel resistance evolution

**Fig 6:**
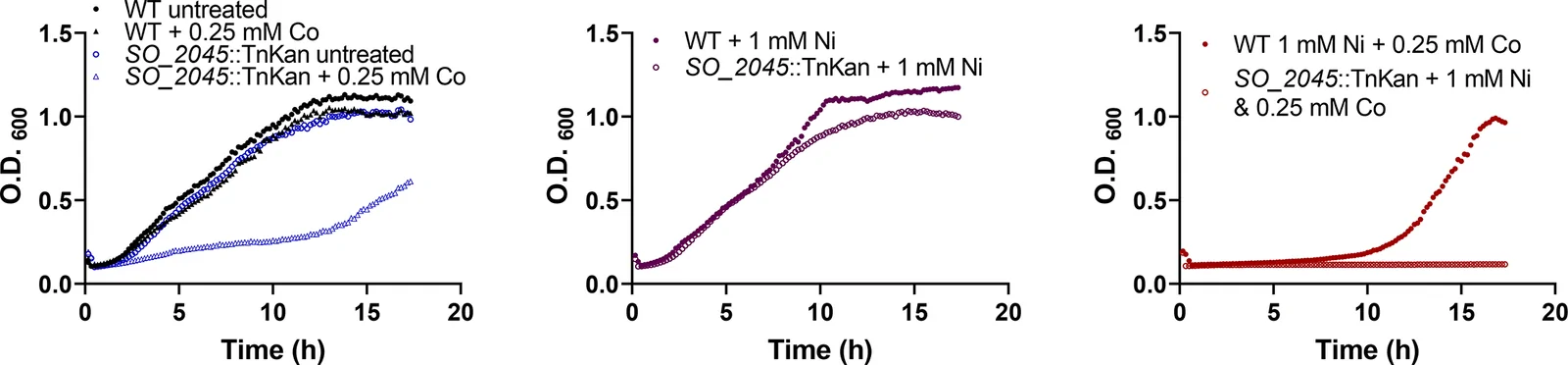
Cobalt and nickel toxicity in CDF-efflux mutant (*SO_2045*::TnKan). Bacterial growth with (**A**) 0.25 mM cobalt, (**B**) 1 mM nickel, and (**C**) the combination of 1 mM nickel and 0.25 mM cobalt in WT and *SO_2045*::TnKan.

Putative annotations of the genes within the duplicated region indicate that one of the amplified genes is a cation diffusion facilitator (CDF) family protein, locus tag *SO_2045*. Because efflux proteins have a well-documented role in metal homeostasis, we were particularly interested in the role of this protein in our system (29, 53, 54). Phylogenetic predictions of *SO_2045* suggest that it is selective for cobalt and nickel metal ions (55). Thus, we sought to determine the role of *SO_2045* in cobalt and nickel resistance evolution by using a *SO_2045* transposon knockout strain obtained from Dr. Buz Barstow (denoted *SO_2045*::TnKan) (56, 57). Optical density measurements of WT and *SO_2045*::TnKan were used to evaluate differences in metal toxicity between the two strains. Cobalt mono-treatment (0.25 mM) in LB media was notably more toxic in the efflux mutant when compared with WT (Fig. 6A). However, sensitivity to 1 mM nickel remained similar between the mutant and WT strains, which suggests that the efflux pump is critical for cobalt tolerance, but nickel tolerance is under the control of an alternate system (Fig. 6B). Additional transcript analysis shows that *SO_2045* is ~100-fold upregulated in metal-treated cells over 48 generations in minimal media relative to passaged controls, further indicating the importance of this gene (Table S7).

We next evaluated the effect of combined cobalt and nickel treatment in the efflux-deficient strain. Growth data reveal that the combination of metal ions is lethal in *SO_2045*::TnKan relative to WT cells (Fig. 6C). These data demonstrate that *SO_2045* has a critical role in *S. oneidensis* adaptation to cobalt toxicity. However, this CDF family efflux protein does not provide insight into the mechanism of cobalt and nickel synergy or why this metal combination specifically induces amplification events. Although we have shown that *SO_2045* has a critical role in metal resistance evolution, future work will involve further analysis of genes beyond the duplicated region to more completely assess the mechanism of cobalt and nickel synergy in *S. oneidensis*.

## DISCUSSION

In this study, we identify a consistent gene amplification event that arises in response to chronic exposure to cobalt and nickel in *S. oneidensis*. A putative CDF-family efflux pump within the 28-kb duplicated region was found to mediate cobalt toxicity, but the intracellular mechanism that drives synergy between cobalt and nickel ions remains unknown. Evidence suggests that CDF-family efflux proteins evolved through gene duplication amplification and are therefore likely to be associated with mobile genetic elements (MGEs) (31). However, amplification of CDF genes has not previously been reported as a mechanism of metal resistance in prokaryotes. We describe a highly dynamic metal-induced gene amplification event in *S. oneidensis* that evolves rapidly, persists for over 1,000 generations in the absence of selection pressure, and can be re-stimulated with stress that does not trigger initial amplification events. The dynamics of this gene amplification suggest transposon-mediated gene duplication; however, additional work is required to fully elucidate the mechanism through which duplications occur (58, 59). Furthermore, in contrast to previous studies that look at gene amplification, we observe that amplification does not cause notable fitness defects in metal-resistant cells. This lack of fitness cost indicates that gene amplification may persist within a microbial community longer than previously thought due to chronic stress. This suggests that gene amplification events should be more widely evaluated as a mechanism of bacterial resistance to environmental toxins and antibiotics.

Chronic exposure to toxins can lead to resistance, which is often mediated by the evolution of and selection for beneficial genetic mutations (60). However, mutations in individual genes evolve slowly, and thus, gene duplication amplification events have been observed as an intermediate, ephemeral response to toxins. The transient nature of gene amplification events is presumed to contribute to heteroresistance, where a subset of the bacterial population is significantly less susceptible to a toxin, but the population does not retain resistance if direct stress is removed (35, 61). Most research indicates that additional copies of genes are rapidly lost in the population in the absence of selection pressure due to the high fitness cost associated with harboring additional DNA. The loss of amplified genes leads to the resensitization of an organism to a given toxin if the organism has not developed a stable, compensatory mutation and drives the transient nature of duplication-mediated resistance. However, the stability of the DNA amplification events presented in this work suggests that amplification may serve as a primary resistance mechanism in some systems and can exist without fitness costs.

Prolonged culturing of metal-resistant cells in the absence of metal stress led to a decrease in the number of amplification events per organism, but in no cases resulted in the complete loss of duplication. It is possible that cells with copy number >1 might have been eliminated from the population if cells were cultured without stress for a longer duration; however, considering that routine cryostress can uniquely elicit additional amplification events in metal-resistant cells, it is unlikely that cells will be able to obtain this prolonged recovery period in a complex environmental matrix. Similarly, organisms maintained ~10 copies throughout prolonged metal exposures even in strains that acquired additional point mutations over the course of treatment. In addition to possessing similar amplification dynamics, strains that developed point mutations had no clear fitness advantage or increased metal resistance compared with strains without mutations. Because cells that acquire additional point mutations do not harbor a competitive advantage over cells that have no additional mutations, we postulate that gene amplification is the primary driver of metal resistance.

Changes in efflux pump expression are known to affect bacterial resistance to both metals and antibiotics (62, 63). Here, we highlight the importance of a putative cation efflux pump, SO_2045, in the evolution of metal resistance in *S. oneidensis*. This efflux pump is a key component of resistance evolution; however, the specific impact of gene copy number on metal tolerance should be pursued in future work. Furthermore, future evaluation of the role of other amplified genes is needed to understand the coordinated response to metal stress observed in *S. oneidensis*.

We demonstrate that this gene amplification event arises as a specific response to cobalt and nickel bimetal stress. To the best of our knowledge, this is the first demonstration of metals interacting synergistically to promote gene amplification events despite the many studies that report bimetallic nanoparticles or dual metal-ion treatments leading to enhanced bacterial inhibition relative to single-metal treatments (64–66). The mechanisms of metal ion synergy remain largely elusive, but it is hypothesized that increased toxicity is due to dual inhibition of intracellular processes, such as DNA damage, membrane integrity, metabolic function, or antioxidant depletion (66, 67). Considering these mechanisms of toxicity, it is not clear why the combination of cobalt and nickel synergizes, while the combination of cobalt and copper or zinc does not. Furthermore, current understanding of mechanisms of metal resistance is largely limited to general stress responses (i.e., efflux, sequestration, and enzymatic conversion) (68). Reversible GDA events in known metal-resistance genes have been observed as a temporary response to individual nickel, copper, or cadmium metal ion stress, but these studies do not address the potential impact of bimetallic stress (69–71). Thus, the intracellular mechanism that elicits gene amplification as a result of specific cobalt and nickel stress in *S. oneidensis* requires further investigation and is of growing importance due to the increasing abundance of cobalt and nickel in the environment (72, 73).

To understand the ecological impact of the evolution of metal resistance in *S. oneidensis*, we must consider the potential for resistance to be shared amongst other organisms. MGEs are critical for horizontal gene transfer (HGT) between bacterial species (74–76). Insertion sequences and transposons can move to new sites on the chromosome or to plasmids within the cell (41). This mobilization is facilitated by transposase enzymes, and thus, the association of resistance genes with transposases and insertion sequences is a critical step in HGT (77–79). Putative functional annotations of the genes duplicated in metal-resistant *S. oneidensis* reveal multiple transposases and integrases that flank approximately 17 cargo genes. Although our long-read sequencing data suggests that the region of interest is primarily inserted locally, the structure of this genomic region and association of cargo genes indicate that this MGE could be exported from *S. oneidensis* and transferred to other organisms in the same environmental niche (78, 80, 81). Furthermore, the MGE may be transferred to a pathogenic species, which is particularly concerning in the context of the growing antibiotic resistance crisis (82–84). Alternatively, the amplified genes may trigger a defense response and lead to the production of novel toxins, which could have deleterious effects on the broader microbial community (85). Further work is needed to elucidate the inter-organism transfer of the MGE studied herein, but it is important to pursue to understand the contribution of gene amplification towards resistance evolution.

The evolution and dissemination of genes that facilitate resistance to environmental toxins is of interest because environmental bacteria and aquatic environments are known to be reservoirs of antibiotic resistance genes (74, 86–89). Indeed, certain antibiotic resistance genes are known to originate from *Shewanella* species (90–92). *S. oneidensis* exists in the microbiome of some aquatic species and coexists with known human pathogens, such as *Vibrio cholerae*, which increases the probability that genes from *S. oneidensis* may be transferred to a pathogen (93). Putative functions of other protein-coding genes within the MGE may provide additional fitness benefits to organisms or protection from environmental toxins not identified in this study. Furthermore, certain genes may be beneficial for survival under antibiotic stress, which underscores the importance of studying gene amplification. Additional functional analysis of the genes amplified in metal-resistant *S. oneidensis* is required to fully categorize the physiological benefits and costs associated with gene amplification and to elucidate the potential impact of sharing these genes with other organisms.

Beyond facilitating cell survival under stress conditions, gene amplification facilitates further genomic adaptation through the potential evolution of novel resistance genes (94–97). Redundancies in the genome provide opportunities for novel mutations to develop without threatening bacterial viability. Thus, the stable gene amplification event presented here may provide a platform for novel resistance to other environmental stressors or antibiotics to arise in *S. oneidensis* and other organisms.

It is known that gene duplication amplifications arise in bacterial populations in response to external stress and that a diverse array of stimuli can promote gene amplification. However, the mechanism through which various stimuli elicit specific duplication events is unknown due to the complexity of intracellular signaling that leads to amplification. Here, we demonstrate that the specific combination of cobalt and nickel is required to elicit amplification in *S. oneidensis*. This suggests that these metal ions have unique intracellular targets that interact to trigger amplification. Additional work is needed to elucidate the specific mechanism through which these metal ions elicit gene amplification.

Future studies will seek to address the ability of genes within the duplicated region to be transferred to other organisms along with developing a more complete understanding of how cobalt and nickel facilitate amplification and changes in biophysical properties brought on by resistance. Together, the work presented here suggests that gene amplification is a more stable mechanism of bacterial resistance than has previously been appreciated. Thus, amplification events should be characterized and considered more thoroughly among resistant populations, as these genomic perturbations may provide an avenue for novel mutations and facilitate the spread of novel antibiotic resistance genes.

## MATERIALS AND METHODS

### DNA extraction and whole genome sequencing

Single colonies were picked from LB agar plates and inoculated into 5 mL of LB. Cultures were grown overnight, diluted to an OD_600_ of 1.0, and 1.8 mL was used for DNA extraction with a Qiagen DNeasy UltraClean Microbial Kit according to manufacturer’s instructions. DNA was eluted in RNase-free water and measured for concentration and purity using a NanoQuant Plate and Tecan Spark Plate reader. DNA was then either submitted for sequencing by SeqCenter (Pittsburgh, PA), or prepared for qPCR copy number analysis described below. For long-read sequencing, cell pellets were shipped to CD Genomics (Shirley, NY), where DNA was extracted using a proprietary method to ensure sufficient read length to capture duplication location in the genome.

### Whole-genome assembly and copy number analysis

Short-read sequencing data were returned as two fastq files per strain corresponding to forward and reverse reads. Reads were mapped and aligned to a reference genome assembly for *Shewanella oneidensis* (GCF_000146165.2). Mapping was done with BWA to produce a .bam file, which was converted to a bigwig file using bamCoverage. All alignment and file conversion were performed via Galaxy (version 24.2). Bigwig files were visualized using integrated genomics viewer (IGV).

Long-read sequencing analyzed from sorted bam files. To identify where duplications were occurring in the genome, data were first filtered for high confidence split reads. Reads were first filtered for the supplementary alignment tag (SA:Z:). Tagged reads were only kept if the primary and supplementary alignments were at least 500 bp apart and both the primary and supplementary alignments had a match of at least 500 bp (with 85% accuracy). To specifically look at the region of interest, we then filtered for reads where the primary or supplementary alignment matched at least 50% of the ROI (with 85% accuracy). Out of the resulting reads, we looked at each alignment to determine if it mapped to a new portion of the genome (>10 kb from the ROI) or was inserted locally, within 10 kb of the ROI. The read counts after data processing are reported in Table S3. The locations of the split reads were then visualized in a dot plot (Fig. S1). Read span and location are represented in the dot plot.

### Determination of transposon copy number by real-time qPCR

DNA concentration was diluted to 10 ng/µL for copy number evaluation. Relative copy number was determined using a qPCR-based method (49). SO_4604 (putative annotation, *sulA*) was selected as the reference gene because there is only one copy in the genome, and it lies outside the duplicated region, which means copy number should be independent of amplifications happening elsewhere in the genome occur. SO_2041 (putative annotation, *rmuC*) was chosen as the target gene. SO_2041 lies within the duplicated region and should thus have a detectable fold change that corresponds to amplification events. Primer sequence are listed in Table S4 of the supplementary information. All copy number assessments were normalized to DNA extracted from the same unpassaged control samples.

### Gene amplification accumulation

*Shewanella oneidensis* MR-1 (ATCC BAA1096) cells streaked on LB agar plates at 30°C. Three colonies were picked and inoculated in 5 mL LB media each for 12 hafter which cells were sub-cultured in technical triplicate. Cells were passaged every 24 generations (~12 h), and aliquots were collected intermittently for DNA extraction at and copy number analysis.

### Gene amplification loss

Three metal-resistant strains were streaked on LB agar plates. Three colonies were picked from each strain and grown in 5 mL pristine LB media. Unperturbed *S. oneidensis* has a doubling time of ~30 min in LB media; thus, there are ~24 generations in a 12-h passage. Cells were diluted 1:250 and passaged every 12 h for 25 days, a total of 1,200 generations (50 passages) in pristine media. Aliquots were taken intermittently for DNA extraction and copy number analysis.

### Metal MIC and synergy

One molar stocks of ZnCl_2_ CuCl_2_ CoCl_2_ NiCl_2_ were prepared in MilliQ H_2_O and sterile filtered. Metal stocks were diluted to appropriate concentrations as follows: 1 mM Ni, Cu, or Zn and 0.25 mM Co. Growth curves were measured in a 96-well plate (in a Tecan Spark microplate reader. Optical density measurements at 600 nm were taken every 10 min. MIC is defined as treatments that yield an optical density less than 0.2 after 20 h of growth in LB media.

## Data Availability

Sequencing data generated in this study can be found in the National Center for Biotechnology Information (NCBI) under accession number PRJNA1468285. All other data produced for this work are contained within the article and its supplemental material.

## SUPPLEMENTAL MATERIAL

Supplemental material aem.01844-25-s0001.pdf Tables S1 to S7; Fig. S1 to S3.

ASM does not own the copyrights to Supplemental Material that may be linked to, or accessed through, an article. The authors have granted ASM a non-exclusive, world-wide license to publish the Supplemental Material files. Please contact the corresponding author directly for reuse.
