# Supplementary material for: Cobalt and nickel ion synergy promotes gene amplification and stable metal resistance in Shewanella oneidensis
Source: Appl Environ Microbiol. 2026 Jul 10;92(8):e01844-25. doi: 10.1128/aem.01844-25 (PMC13488326; doi:10.1128/aem.01844-25)
Supplement: Supplementary file 1 [file aem.01844-25-s0001.pdf]

Supporting Information for

Cobalt and nickel ion synergy promotes gene amplification and stable metal resistance in *Shewanella oneidensis*

Alessandra G. Gavin[a], Stephanie L. Mitchell[a], and Erin E. Carlson[a,b,c,d]*

[a]*Department of Chemistry, University of Minnesota, 207 Pleasant Street SE, Minneapolis, Minnesota 55455, United States*

[b]*Department of Medicinal Chemistry, University of Minnesota, 208 Harvard Street SE, Minneapolis, Minnesota 55454, United States*

[c]*Department of Biochemistry, Molecular Biology, and Biophysics, University of Minnesota, 321 Church St SE, Minneapolis, Minnesota 55454, United States*

[d]*Department of Pharmacology, University of Minnesota, 321 Church St SE, Minneapolis, Minnesota 55454, United States*

List of Tables

Table S1. Mutations in metal- and NMC-resistant *S. oneidensis* identified through whole-genome sequencing.

Table S2. Putative annotations of duplicated genes.

Table S3. Long-read sequencing read counts for local and distal split reads mapping to the ROI.

Table S4. Sequences used for copy number qPCR.

Table S5. Mutations identified in metal-resistant cells after 1,128 generations in metal-free LB.

Table S6. Mutations identified in cells after 1,128 generations with metals in LB.

Table S7. Transcript analysis of SO_2045 after 48 generations of metal-treatment. Log_2 fold change relative to passaged control.

List of Figures

Figure S1. Dot plot visualization of split reads in long-read sequencing data.

Figure S2. Copy number analysis upon 35 μM single-ion metal treatment.

Figure S3. Bacterial growth after 120 generations of metal exposure.

Table S1. Mutations identified in whole-genome sequencing of metal- and nanomaterial-resistant *S. oneidensis.*

Strain	Location	Locus	Original	Mutation	Gene
Metal-resistant	Plasmid	38,067	ATTTTTTTAT	ATTTTTTTTATG, insertion	SO_A0176, hypothetical
NMC-resistant	Chromosome	256,847	G	C	*rpsK*, SO_0254
NMC-resistant	Chromosome	257,910	T	C	*rpoA*, SO_0256
NMC-resistant	Chromosome	259, 251	G	A	Non-coding
NMC-resistant	Chromosome	865,439		TCAGCAT insertion	Non-coding
NMC-resistant	Chromosome	1,487,743	G	A	Non-coding
NMC-resistant	Chromosome	3,388,411		GTCATACGGTAGAG deletion	*flgB*, SO_3250
NMC-resistant	Chromosome	4,902,469	C	A	SO_4700

Table S2. Putative annotations of genes identified within the duplicated region in metal- and nanomaterial-resistant *S. oneidensis*.

Locus Tag	Gene	Putative Annotation	Cellular location
SO_2032	tnpA	ISSod1 transposase	Cytosol
SO_2034		Hypothetical protein	
SO_2035	tnpA	ISSod25 transposase	Inner membrane
SO_2036		ISSod25 integrase Int_ISSod25	cytosol
SO_2037	intl	Integron integrase	cytosol
SO_2039		Signaling protein with EAL domain	Inner membrane
SO_2040	sltY	Soluble lytic murein transglycosylase	periplasm
SO_2041	rmuC	DNA recombination protein	cytosol
SO_2042	yedY	Oxidoreductase molybdenum-binding subunit	
SO_2043	yedZ	Oxidoreductase cytochrome b subunit	Inner membrane
SO_2044	gloA	Lactoylglutathione lyase	cytosol
SO_2045		Cation efflux protein CDF family	Inner membrane
SO_2046		Transcriptional regulator MarR family	cytosol
SO_2047		Prolyl oligopeptidase family S9 protein	periplasm
SO_2048		Phosphoethanolamine transferase	Inner membrane
SO_2049		Diguanylate cyclase	cytosol
SO_2050		Methylase	cytosol
SO_2051		Hypothetical protein	
SO_2052		FAD-dependent oxidoreductase	cytosol
SO_2053		Transcriptional regulator LysR family	cytosol
SO_2054	frmA	S-(hydroxymethyl)glutathione dehydrogenase	cytosol
SO_2055	frmB	S-formylglutathione hydrolase	
SO_2057	tnpA	ISSod1 transposase	

Table S3. Long-read sequencing read counts for local and distal split reads mapping to the ROI

	CNR 1	CNR 2	CNR 3	WT
High-confidence split reads*	18277	12642	29389	13909
Split reads with footprint in > 50% ROI**	601	112	521	9
Distal translocations	7	5	5	2
Local tandems	471	90	414	7

** High-confidence split reads are defined as reads that have a supplementary alignment tag (SA:Z:) such that both the primary and supplementary alignments have a match of at least 500 bp.*

*** Split reads with footprint in >50% ROI are defined as reads that match the high-confidence split read condition and cover at least 50% of the region of interest (ROI) - genomic coordinates 2128886-2157241.*

Table S4. Primer sequences used for copy number qPCR

Target gene symbol	Direction	Sequence (5' to 3')	Amplicon size	T_m	Reference
rmuC SO_2041	Forward	TCAAATTGGGTTTGCAGGCG	184	59.97	This study
rmuC, SO_2041	Reverse	TGCCGAGTTTAGAGCAGCAA	184	59.97	This study
sulA, SO_4604	Forward	TGTTCGGGACGCAGATAGTG	250	59.83	This study
sulA, SO_4604	Reverse	TTCCATTGCCCACAGGGTTT	250	60.11	This study

Table S5. Mutations identified in metal-resistant cells after 1,128 generations in metal-free LB.

Strain	Location	Locus	Original	Mutation	Gene
Prolonged recovery (LB) **1**	Chromosome	1,699,961	A	G	Intergenic, *tnpA 38* (SO_1616) *,* SO_1617
Prolonged recovery (LB) **1**	Chromosome	2,832,134	C	G	Intergenic, *pnuT* (SO_2713), *ykoF* (SO_2174)
Prolonged recovery (LB) **2**	Chromosome	3,153,903	A	G	Intergenic, SO_3040, SO_3043
Prolonged recovery (LB) **2**	Chromosome	3,153,916	G	T	
Prolonged recovery (LB) **2**	Chromosome	3,153,943	G	A	
Prolonged recovery (LB) **2**	Chromosome	3,153,949	T	G	
Prolonged recovery (LB) **2**	Chromosome	3,153,988	G	A	
Prolonged recovery (LB) **2**	Chromosome	3,153,026	T	A	
Prolonged recovery (LB) **3**	Chromosome	2,689,107	C	T	SO_2557

Table S6. Mutations identified in cells after 1,128 generations with metals in LB.

Strain	Location	Locus	Original	Mutation	Gene

Prolonged metal exposure (LB) **1**	Chromosome	650,960	C	T	*crp*, SO_0624
Prolonged metal exposure (LB) **1**	Chromosome	1,162,400		TTT insertion	Intergenic, SO_1117, SO_1120
Prolonged metal exposure (LB) **2**	*No mutations identified*				
Prolonged metal exposure (LB) **3**	Chromosome	1,386,400	C	T	*cyaC*, SO_1329
Prolonged metal exposure (LB) **3**	Chromosome	2,047,497	C	G	SO_1944
LB passaged control	*No mutations identified*				

Table S7. Transcript analysis of SO_2045 after 48 generations of metal-treatment. Log_2 fold change relative to passaged control.

**Gene**	**Log_2 Fold Change**	**Adjusted p value**
SO_2045	6.01714	2.02 E-55

A)

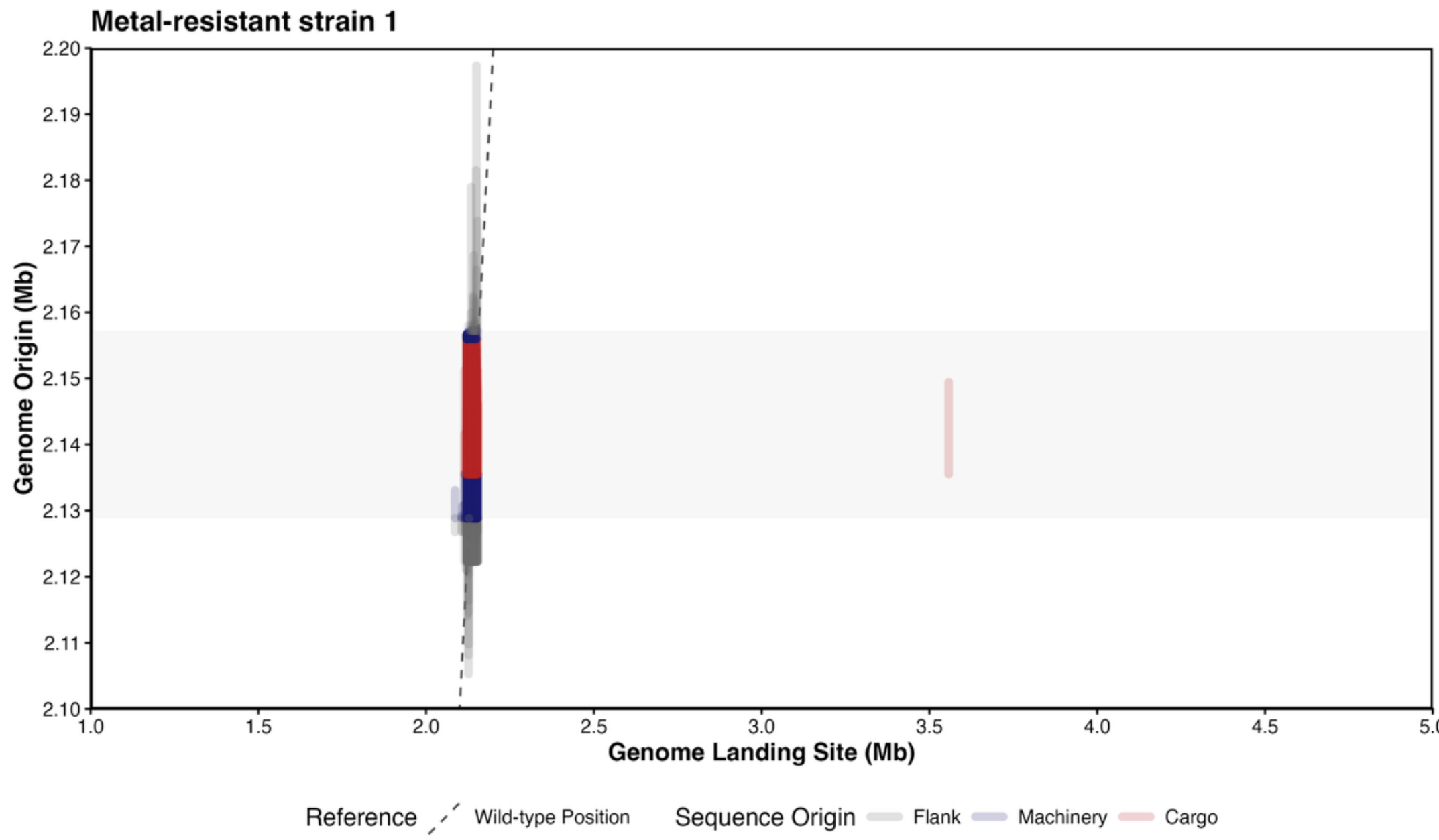
Metal-resistant strain 1
Genome Origin (Mb)
2.20
2.19
2.18
2.17
2.16
2.15
2.14
2.13
2.12
2.11
2.10
1.0
1.5
2.0
2.5
3.0
3.5
4.0
4.5
5.0
Genome Landing Site (Mb)
Reference
Wild-type Position
Sequence Origin
Flank
Machinery
Cargo

B)

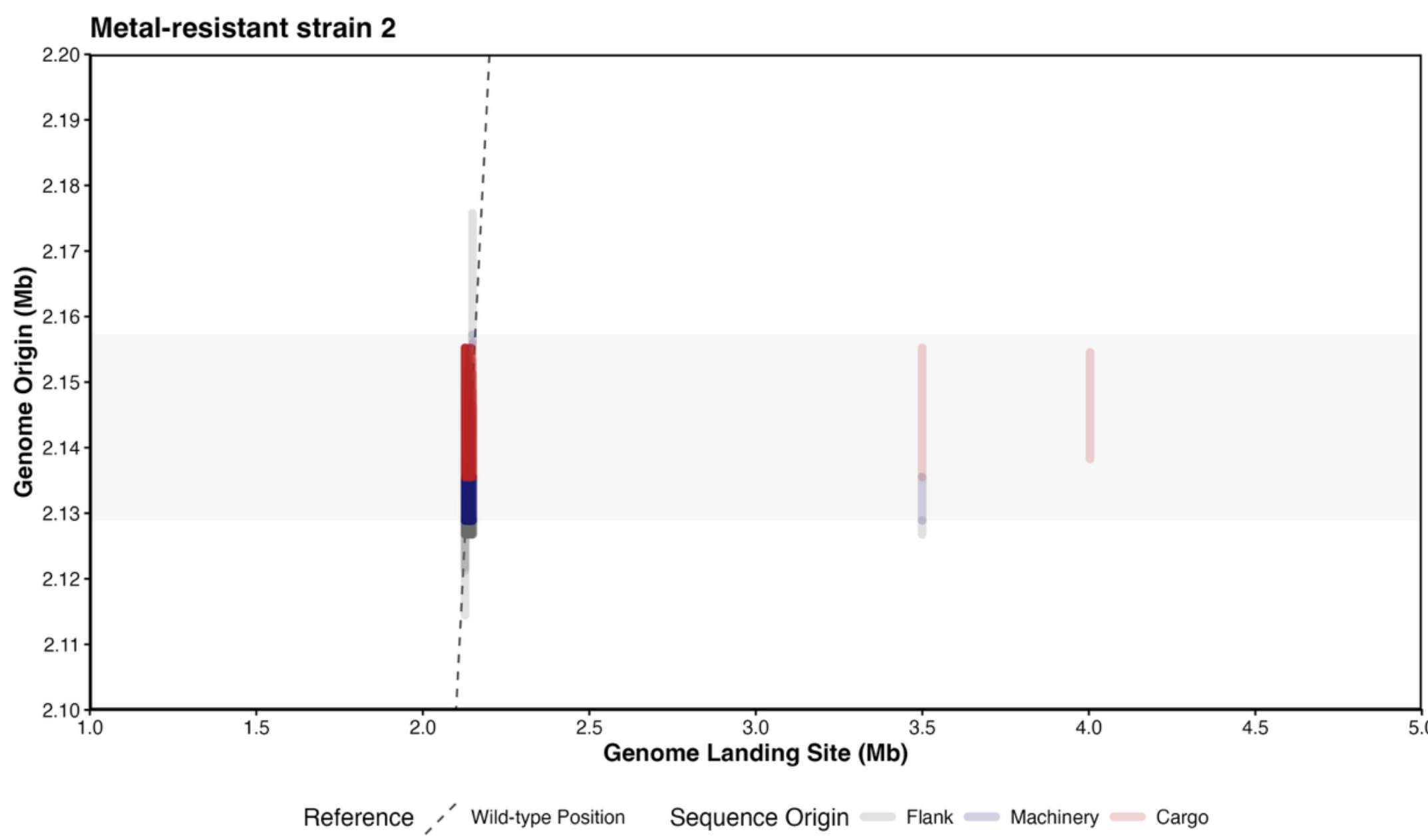
Metal-resistant strain 2
Genome Origin (Mb)
2.20
2.19
2.18
2.17
2.16
2.15
2.14
2.13
2.12
2.11
2.10
1.0
1.5
2.0
2.5
3.0
3.5
4.0
4.5
5.0
Genome Landing Site (Mb)
Reference
Wild-type Position
Sequence Origin
Flank
Machinery
Cargo

C)

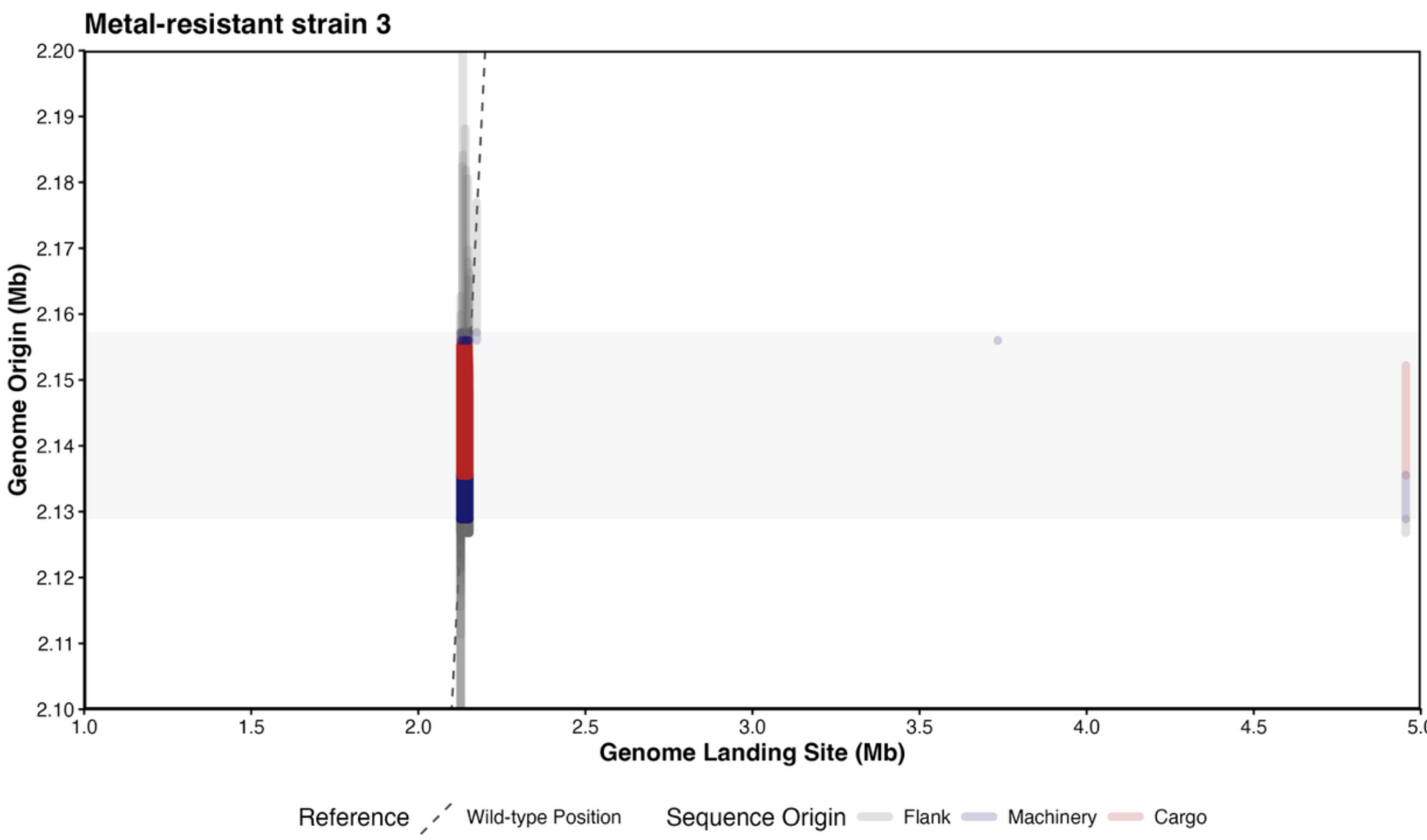

D)

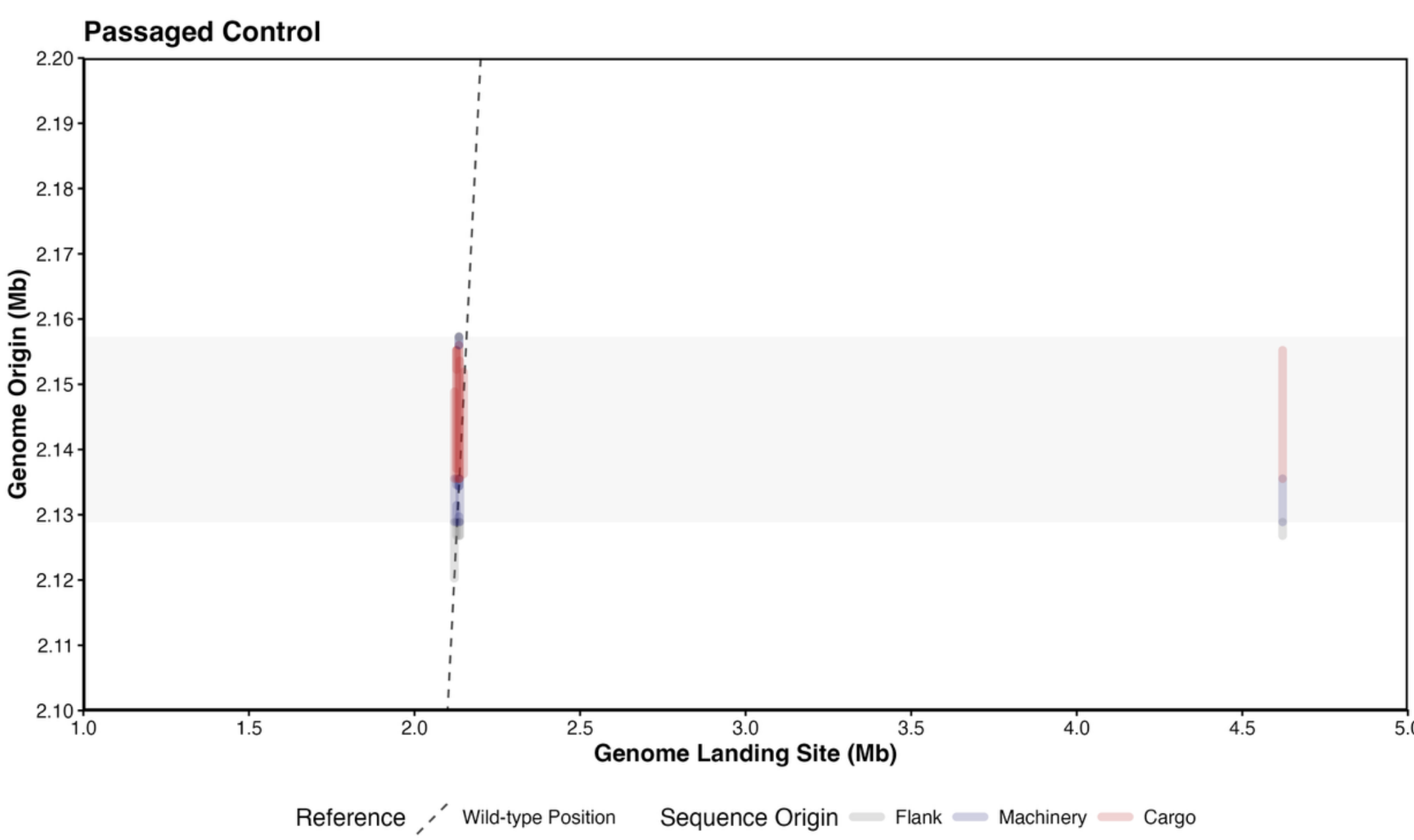

Figure S1. Dot plot visualization of split reads in long-read sequencing data. A-C) Split reads identified in three metal-resistant strains. D) Split reads identified in our passaged control. Location along the x-axis represents the genomic location of either the portion of the read that does not map to the ROI (distal translocations) or the supplementary alignment (local tandems). The y axis represents the span of the ROI (light grey shading). Portions of the read that map to cargo genes are in red, transposition machinery are blue, and flanking regions of the genome are grey. Read density is represented by color transparency.

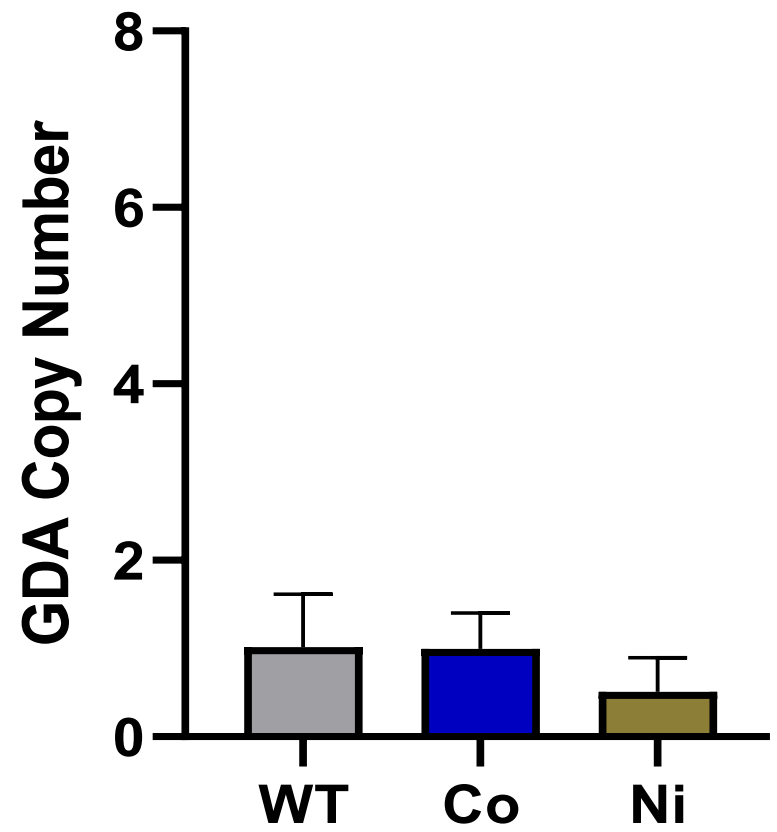

Figure S2. Copy number analysis after 48 generations of 35 µM single-ion metal treatment compared to passaged control (WT).

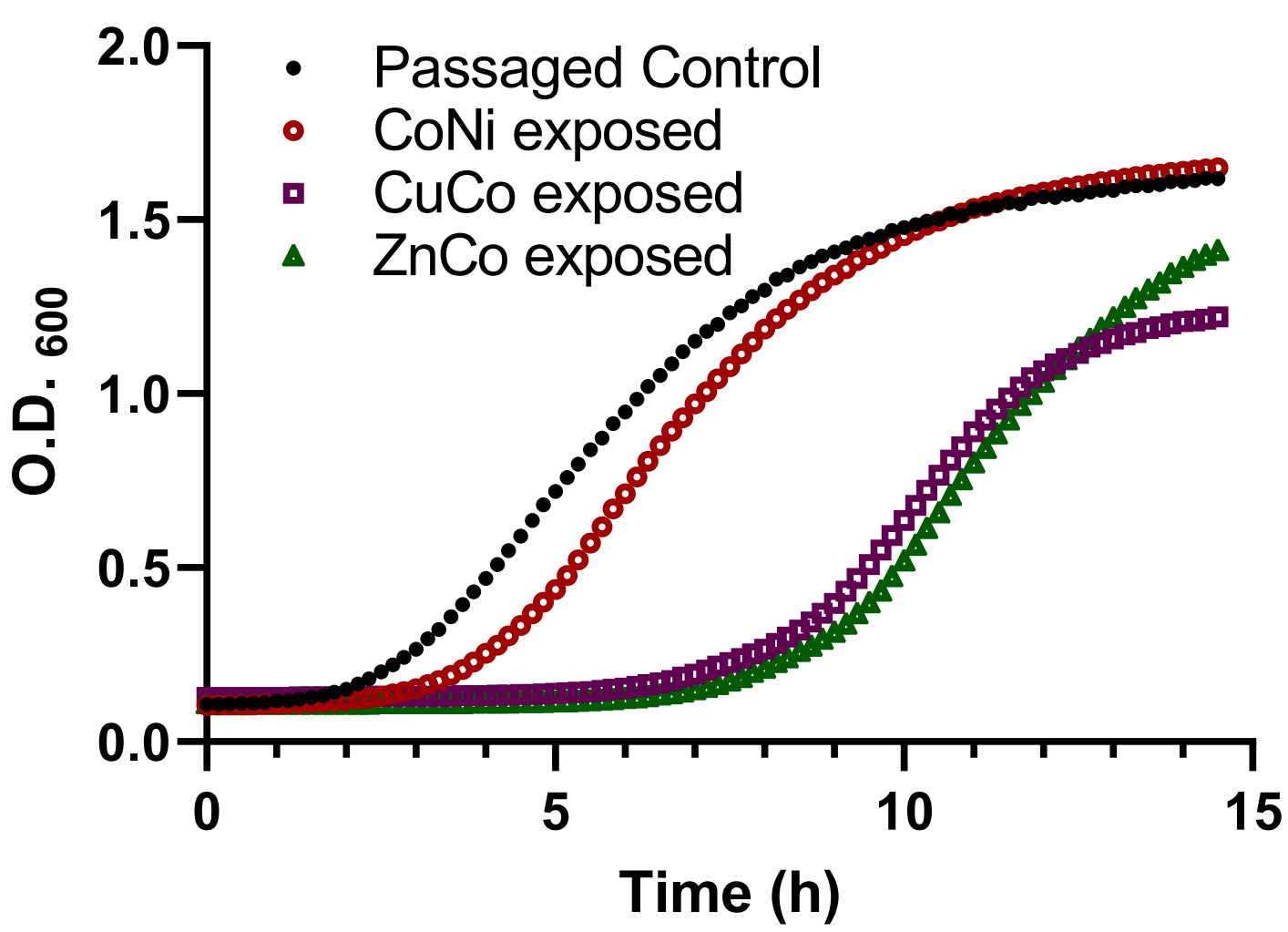

Figure S3. Bacterial growth analysis after 120 generations of exposure to either Cu and Co, Zn and Co, or Ni and Co.
